# Inhibition of Cell Proliferation and Growth of Pancreatic Cancer by Silencing of Carbohydrate Sulfotransferase 15 *In Vitro* and in a Xenograft Model

**DOI:** 10.1371/journal.pone.0142981

**Published:** 2015-12-07

**Authors:** Kazuki Takakura, Yuichiro Shibazaki, Hiroyuki Yoneyama, Masato Fujii, Taishi Hashiguchi, Zensho Ito, Mikio Kajihara, Takeyuki Misawa, Sadamu Homma, Toshifumi Ohkusa, Shigeo Koido

**Affiliations:** 1 Division of Gastroenterology and Hepatology, Department of Internal Medicine, The Jikei University School of Medicine, Tokyo, Japan; 2 Stelic Institute & Co., Inc, Tokyo, Japan; 3 Department of Surgery, The Jikei University School of Medicine, Tokyo, Japan; 4 Department of Oncology, Institute of DNA Medicine, The Jikei University School of Medicine, Tokyo, Japan; 5 Institute of Clinical Medicine and Research, The Jikei University School of Medicine, Kashiwa City, Chiba, Japan; University of Nebraska Medical Center, UNITED STATES

## Abstract

Chondroitin sulfate E (CS-E), a highly sulfated glycosaminoglycan, is known to promote tumor invasion and metastasis. Because the presence of CS-E is detected in both tumor and stromal cells in pancreatic ductal adenocarcinoma (PDAC), multistage involvement of CS-E in the development of PDAC has been considered. However, its involvement in the early stage of PDAC progression is still not fully understood. In this study, to clarify the direct role of CS-E in tumor, but not stromal, cells of PDAC, we focused on carbohydrate sulfotransferase 15 (CHST15), a specific enzyme that biosynthesizes CS-E, and investigated the effects of the CHST15 siRNA on tumor cell proliferation *in vitro* and growth *in vivo*. CHST15 mRNA is highly expressed in the human pancreatic cancer cell lines PANC-1, MIA PaCa-2, Capan-1 and Capan-2. CHST15 siRNA significantly inhibited the expression of CHST15 mRNA in these four cells *in vitro*. Silencing of the CHST15 gene in the cells was associated with significant reduction of proliferation and up-regulation of the cell cycle inhibitor-related gene p21^CIP1/WAF1^. In a subcutaneous xenograft tumor model of PANC-1 in nude mice, a single intratumoral injection of CHST15 siRNA almost completely suppressed tumor growth. Reduced CHST15 protein signals associated with tumor necrosis were observed with the treatment with CHST15 siRNA. These results provide evidence of the direct action of CHST15 on the proliferation of pancreatic tumor cells partly through the p21^CIP1/WAF1^ pathway. Thus, CHST15-CS-E axis-mediated tumor cell proliferation could be a novel therapeutic target in the early stage of PDAC progression.

## Introduction

Pancreatic ductal adenocarcinoma (PDAC) has firmly been established as one of the most lethal solid human tumors worldwide [1]. PDAC-related morbidity and mortality cases have both shown to be increasing. Investigation of the mechanisms underlying the malignant characteristics of PDAC, including late diagnosis, aggressive invasion, early dissemination and chemo-resistance, is urgently needed to establish effective treatment and to improve prognosis. Tumor progression involves a multi-step process, such as proliferation, invasion, metastasis and angiogenesis, yet, these processes remain poorly understood in PDAC. In primary pancreatic cancer cells, proliferative signals are kept constitutively active by mutant genes, and the infinite proliferation of genetically distinct sub-clones is observed before the invasion process. Genetic alterations could occur at distant sites after metastasis [2–4]. All stages of tumor progression are also affected by the surrounding microenvironment where glycan plays a pivotal role in the modification of tumor cells and in genetic mutations. The tumors contain various glycosaminoglycans (GAGs) that regulate cell behaviors by interacting with different molecules such as growth factors, cytokines, chemokines, proteinases and their inhibitors [5]. GAGs include chondroitin sulfate (CS), heparin sulfate, keratan sulfate, and hyaluronic acid. The sugar backbone of CS consists of repeating disaccharide units of D-glucuronic acidβ1-3*N*-acetyl-D-galactosamine (GalNAc). During chain elongation in the biosynthesis of CS, the disaccharide units are modified by specific sulfotransferases at C-2 of GlcA and C-4 and/or C-6 of GalNAc in various combinations and display enormous structural diversity, producing characteristic sulfate patterns critical for binding to a variety of functional proteins. Highly sulfated disaccharide units like E-unit, GlcAβ1-3GalNAc (4S, 6S), where 4S and 6S stand for 4-*O*- and 6-*O*-sulfate, respectively, and E-unit-rich CS (CS-E) preparations are synthesized by a specific enzyme, carbohydrate sulfotransferase 15 (CHST15), and show remarkable biological activities [6–12]. Accumulating evidence has revealed the involvement of CS-E in tumor cell invasion and metastasis in the lung [6, 13], ovary [7, 14], breast [15] and skin [16]. The role of CS-E in initiating tumor cell invasion was demonstrated, suggesting the potential of CS-E as a key molecule in establishing novel therapeutics against various solid tumors including PDAC [17].

CS-E is expressed in both the tumor cells and stromal cells surrounding the tumor in PDAC patient tissues [17, 18]. Given the distribution pattern of CS-E, the multistage and multicellular involvement of CS-E in PDAC progression has been considered. A stepwise investigation is therefore needed to reveal the precise mechanisms of CS-E in the underlying malignant characteristics of PDAC. However, the involvement of CS-E in preliminary PDAC progression has yet to be explored. In this study, to investigate whether CS-E functions directly in the proliferation of PDAC or not, we conducted blocking experiments using small interfering RNA designed to inhibit the expression of CHST15 (CHST15 siRNA) that selectively inhibit CS-E biosynthesis. In simple *in vitro* and *in vivo* xenograft experiments, we demonstrate the effect of CHST15 siRNA on the proliferation of PDAC and discuss the potential of CS-E as a therapeutic target for PDAC.

## Materials and Methods

### Materials and reagents

CHST15 siRNA was purchased from Ambion, Inc. (TX, USA). CS-E was obtained from SEIKAGAKU CORPORATION (Tokyo, Japan), reconstituted in phosphate buffered saline, aliquoted and stored at -20°C. WST-1 was obtained from Roche Diagnostic GmbH (Mannheim, Germany). Lipofectamine^™^ RNAiMAX Reagent and Invivofectamine^®^ 2.0 Reagent were purchased from Invitrogen (CA, USA).

### Cell lines and mice

PANC-1, MIA PaCa-2, Capan-1 and Capan-2, pancreatic cancer cell lines, were purchased from the ATCC (Rockville, MA, USA). After checking that the cells were free of mycoplasma infection using the Mycoplasma PCR ELISA kit (Roche Diagnostics, Mannheim, Germany), PANC-1 and MIA PaCa-2, cells were cultured in DMEM (SigmaAldrich, CA) containing 10% fetal calf serum (FCS) and 1% antibiotics. Capan-1 and Capan-2 cells were cultured in IMDM (Wako Pure Chemical Industries, Osaka, Japan) and McCoy’s 5A Medium Modified (Sigma-Aldrich, CA) containing 10% FCS and 1% antibiotics, respectively. Cells were maintained at 37°C in a humidified atmosphere of 5% CO2/air.

### Transfection of siRNA

RNA interference (RNAi) was performed using the reverse transfection method: prior to cell plating, siRNA (50 nmol), Lipofectamine 2000 and Opti-MEM (Invitrogen, Karlsruhe, Germany) media were mixed and incubated according to the manufacturer’s instructions. After transfection for 48 h, cells were collected and used for the other analyses.

### Real time semi-quantitative RT-PCR

Total RNA was extracted using the SV Total RNA Isolation System (Promega, WI, USA) according to the manufacturer’s instructions. RNA yield and purity were determined by spectrophotometry. Reverse transcription was performed with Moloney Murine Leukemia Virus Reverse Transcriptase (Invitrogen) and random hexamers (Promega, WI). Real-time RT-PCR was performed using SYBR Green using the DICE thermal cycler according to the manufacturer's instructions (TAKARA BIO INC., Otsu, Japan). All PCR primers were designed and synthesized by TAKARA BIO INC. Gene expression levels were normalized to glyceraldehyde-3-phosphate dehydrogenase (GAPDH) and presented as arbitrary units. The sense and antisense primers used were shown in Table 1. And the primer sequences are listed in Table 2. Independent experiments were repeated three times for each sample, and the relative expression levels of genes were analyzed.

**Table 1:** Target primer of CHST15 gene.

siRNA	siRNA target primer
CHST15 siRNA	Anti-Sense primer: 5’-UCAAUCCUAGUUGUGAUGCTG-3’
	Sense primer: 5’-GCAUCACAACUAGGAUUGATT-3'
Negative control siRNA	Anti-Sense primer: 5’-UAC GUA CUA UCG CGC GCG GAU-3’
	Sense primer: 5’-AUC CGC GCG CGA UAG UAC GUA-3’

**Table 2:** Sequences of primer for RT-PCR.

Genes	Sequences
CHST15	F: 5’- GCCACTCAATGCCATCCAGA-3’
	R: 5’- ATGGCAGGCTCGAGAACCAC-3’
GAPDH	F: 5’- GCACCGTCAAGGCTGAGAAC-3’
	R: 5’- TGGTGAAGACGCCAGTGGA-3’
p21	F: 5’- TCAAATCGTCCAGCGACCTTC-3’
	R: 5’-CATGCCCTGTCCATAGCCTCTAC-3’

F: forward; R: reverse

### WST-1 cell proliferation assay

For the cell proliferation assay, PANC-1, MIA PaCa-2, Capan-1 and Capan-2 cells transfected with CHST15 siRNA or negative control-siRNA were seeded in a 96-well plate at a concentration of 1 × 10^4^ cells/well. Cellular proliferation was examined after 60 minutes from the start of incubation with the cell proliferation reagent WST-1 (Roche Molecular Biochemicals, Mannheim, Germany).

### Cell proliferation assay with HGF and CS-E

PANC-1 cells (1,000 cells) were seeded in culture medium in a 96-well plate the day before HGF stimulation. The cells were stimulated with 5 ng/mL HGF with or without 100 μg/mL CS-E, and cell proliferation was determined using the WST-1 assay for 120 min at 37°C.

### CHST15 siRNA treatment in a PANC-1 xenograft model

BALB/c nude mice (6 to 9 weeks old) were purchased from CLEA-Japan (Tokyo, Japan) and were maintained under specific pathogen-free conditions. This study was carried out in strict accordance with the recommendations in the Guide for the Care and Use of Laboratory Animals of the National Institutes of Health. The protocol was approved by the Committee on the Ethics of Animal Experiments of The Jikei University School of Medicine (Permit Number: 25–067). All surgery was performed under sodium pentobarbital anesthesia, and all efforts were made to minimize suffering. PANC-1 cells (1 × 10^7^ cells) in 100 μL of BD Matrigel^™^ Matrix (BD, NJ) were injected subcutaneously into both flanks of nude mice [19]. Intratumoral injection of either 100 μL of 250 nM control siRNA or CHST15 siRNA complexed with Invivofectamine^®^ 2.0 Reagent (Life Technologies, CA) was performed 7 days after the inoculation. Tumor size was measured every other day. On day 9 and day 14, the mice were sacrificed, and the tumors were isolated, weighed and used for gene expression or histological analyses.

### Histological analysis

Tumor tissues were fixed in 10% phosphate-buffered formalin. After fixation, the tissues were embedded in paraffin, and cut slides were used for Hematoxylin-Eosin (HE) staining and immunohistochemical staining as described previously [20] for CHST15 using an anti-human CHST15 antibody (Sigma-Aldrich) at a dilution of 1:25. As a secondary antibody, Dako EnVision^™^ FLEX/HRP detection reagent (Dako, Denmark) was used. No positive signal was detected when omitting first antibody, supporting the specificity of the staining (data not shown).

### Statistical analysis

All data are presented as the mean ± standard deviation (SD). Statistical analyses were performed using one-way ANOVA followed by the Bonferroni correction or Student’s t-test. A *p* value of less than 0.05 was considered statistically significant.

## Results

### Target-specific siRNA-induced CHST15 knockdown in pancreatic cancer cell line

We first confirmed the abundant expression of CHST15 mRNA in the pancreatic cancer cell lines PANC-1, MIA PaCa-2, Capan-1 and Capan-2 using RT-PCR. Next, these four cells were transfected with either target-specific siRNA duplexes (CHST15 siRNA) or a non-specific siRNA control (control siRNA). Gene silencing was measured by real-time RT-PCR, and CHST15 expression was reduced in PANC-1 cells by 1%, 85% and 87% in samples treated with target-specific siRNA for 24 h, 48 h and 72 h, respectively. At 48 h after the transfection, dose-dependent suppression of CHST15 mRNA expression levels was observed (data not shown). Therefore, in successive studies, we investigated the effect of CHST15 siRNA 48 h after siRNA transfection. CHST15 expression was reduced in MIA PaCa-2, Capan-1 and Capan-2 cells by 75%, 42% and 36% in samples treated with target-specific siRNA for 48 h, respectively.

### Inhibition of pancreatic cancer cell proliferation induced by CHST15 siRNA

**Fig 1:**
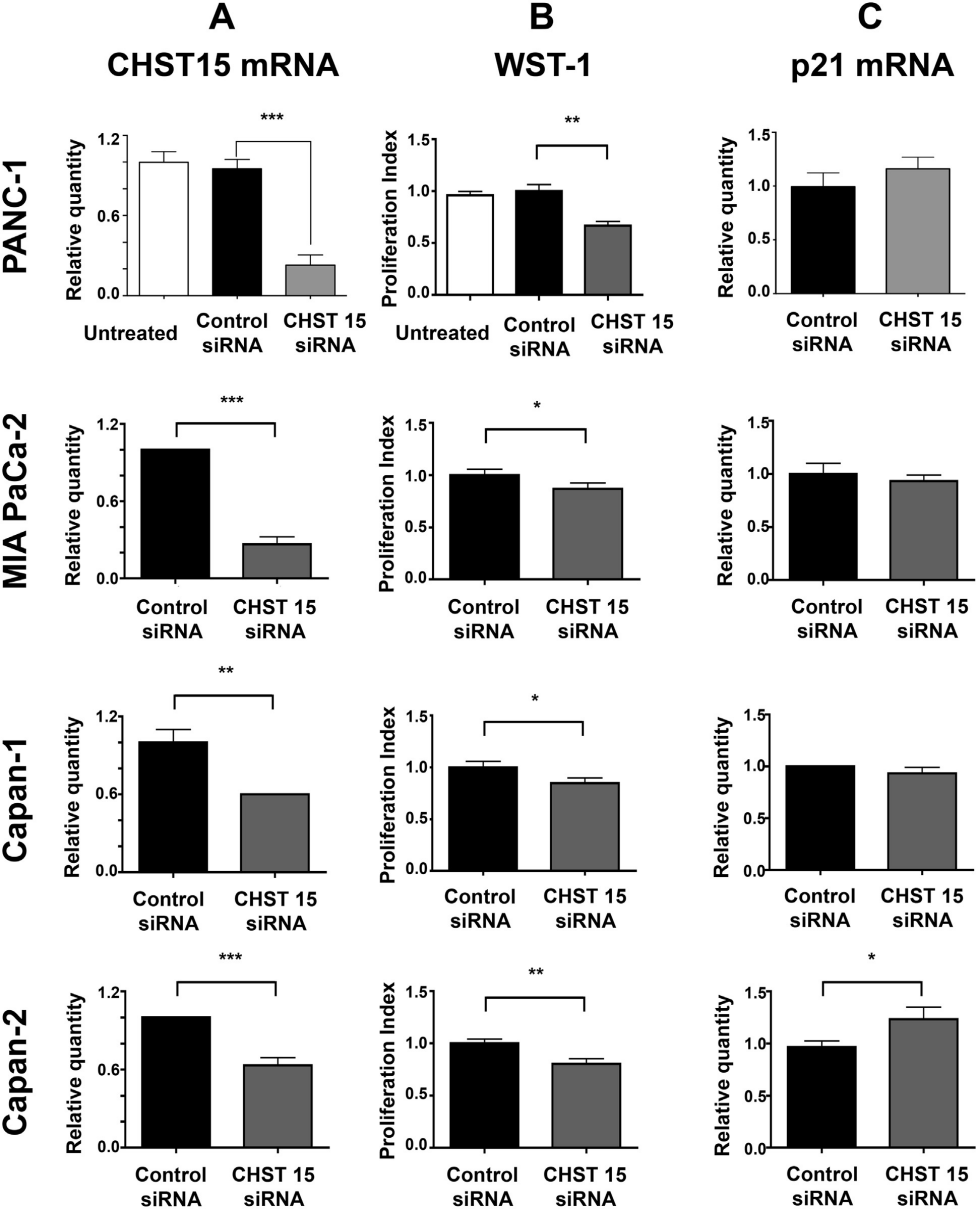
The knockdown effect of CHST15 siRNA on pancreatic cancer cell lines. (A) Relative quantities of CHST15 mRNA in control siRNA treated and CHST15 siRNA treated PANC-1, MIA PaCa-2, Capan-1 and Capan-2 cells (n = 3 in each) were shown after normalization using GAPDH as an internal control. In each cell lines, there were significant differences about the reduction of CHST15 expression in samples treated with target-specific siRNA for 48 h. Asterisks (**, ***) p<0.01, P<0.001, respectively between control siRNA and CHST15 siRNA. (B) A WST-1 assay. The mean level of proliferation in control siRNA treated cells (black column) was expressed as a standard (1.0) and the data were shown relative to the standard as a proliferation index. Mean±SD (n = 3). Significant suppressive effects of CHST15 siRNA on cell proliferation were observed in PANC-1, MIA PaCa-2, Capan-1 and Capan-2. Asterisks (*, **) p<0.05, P<0.01, respectively between control siRNA and CHST15 siRNA. (C) Relative quantities of p21 mRNA in control siRNA treated and CHST15 siRNA treated PANC-1 cells (n = 3–5 in each). There was a slight increase and a significant increase of p21 mRNA in CHST15 siRNA treated PANC-1 cells and Capan-2 cells compared to control siRNA, respectively. Asterisk (*) p<0.05 between CHST15 siRNA and control siRNA.

To assess the effect of CHST15 knockdown (Fig 1A), cell proliferation was examined 48 h after the start of siRNA transfection using the WST-1 assay. The results showed the significant suppressive effect of CHST15 siRNA on cell proliferation in PANC-1, MIA PaCa-2, Capan-1 and Capan-2 (Fig 1B). An increase in p21 gene expression levels was detected in CHST15 siRNA-treated cells compared to the control siRNA-treated cells in PANC-1and Capan-2 (Fig 1C).

### Effect of CHST15 siRNA on pancreatic tumor growth in a xenograft model

**Fig 2:**
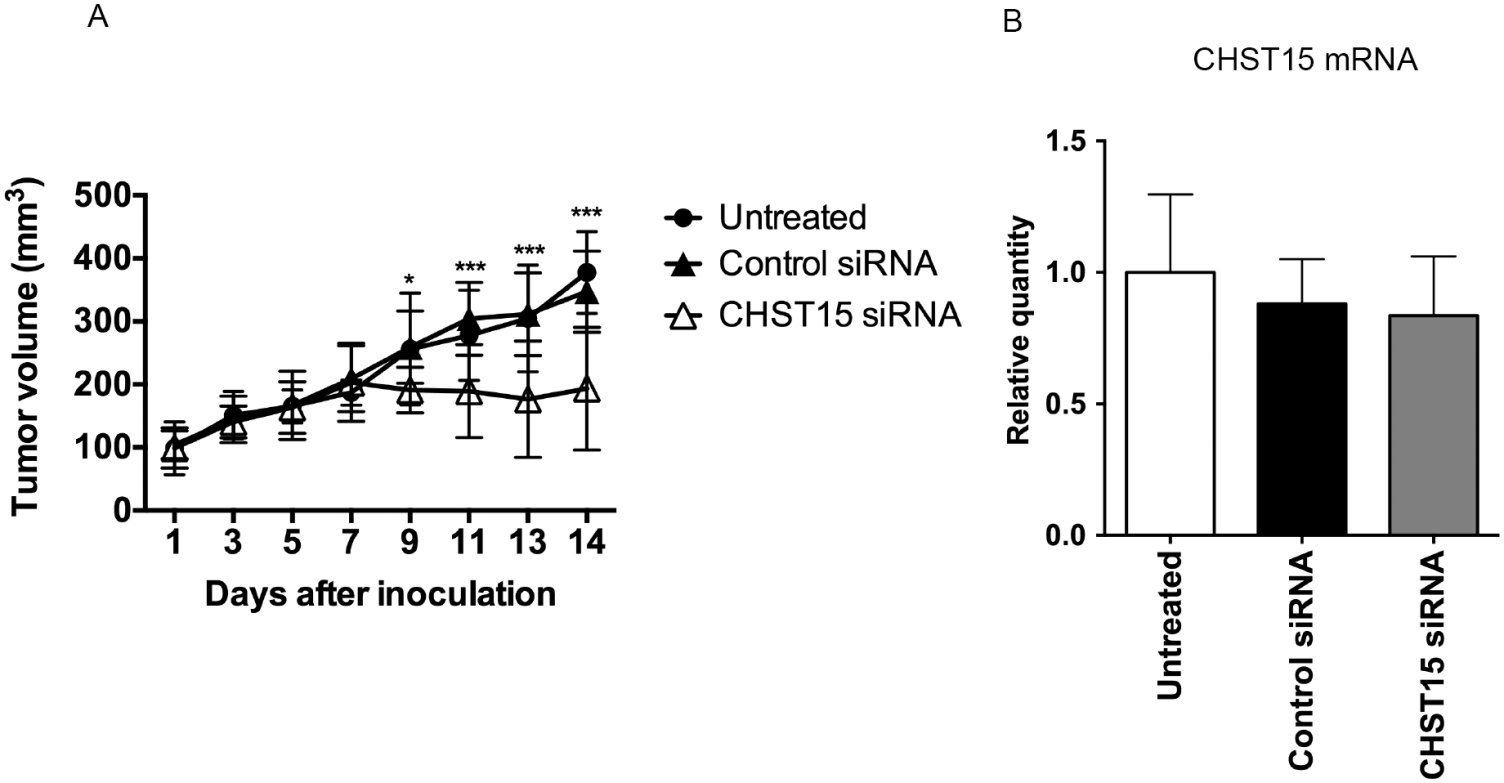
Effect of CHST15 siRNA on tumor growth in a xenograft model. (A) The change of tumor volume. Data were expressed as mean ± SD (Untreated; n = 6, Control siRNA: n = 12, CHST15 siRNA; n = 10). Asterisks (*, ***) p<0.05, P<0.001, respectively between control siRNA and CHST15 siRNA. (B) Relative quantities of CHST15 mRNA in untreated, control siRNA treated and CHST15 siRNA treated xenografts at day 14. Data were expressed as mean±SD (Untreated; n = 6, Control siRNA: n = 12, CHST15 siRNA; n = 10).

After confirming PANC-1 cell growth in the nude mice on day 7 after inoculation, we investigated the suppressive effect of CHST15 siRNA on PANC-1 cell proliferation *in vivo* (Fig 2). After injecting PANC-1 cells in both flanks of nude mice, we administered CHST15 siRNA into each tumor on day 7. The anti-proliferative effect of CHST15 siRNA was identified against control siRNA after day 9. PANC-1 cells treated with CHST15 siRNA grew more slowly than control siRNA-treated cells and non-treated cells (Fig 2A). These results indicate that the proliferation of PANC-1 cells was suppressed following the knockdown of CHST15. Real time RT-PCR was performed to assess the gene-suppressive effect, but there was no significant difference in gene expression between CHST15 siRNA-treated and control siRNA-treated mice either on day 9 (data not shown) or day 14 (Fig 2B).

### Pathological examination of pancreatic tumor by hematoxylin and eosin staining and immunohistochemistry

**Fig 3:**
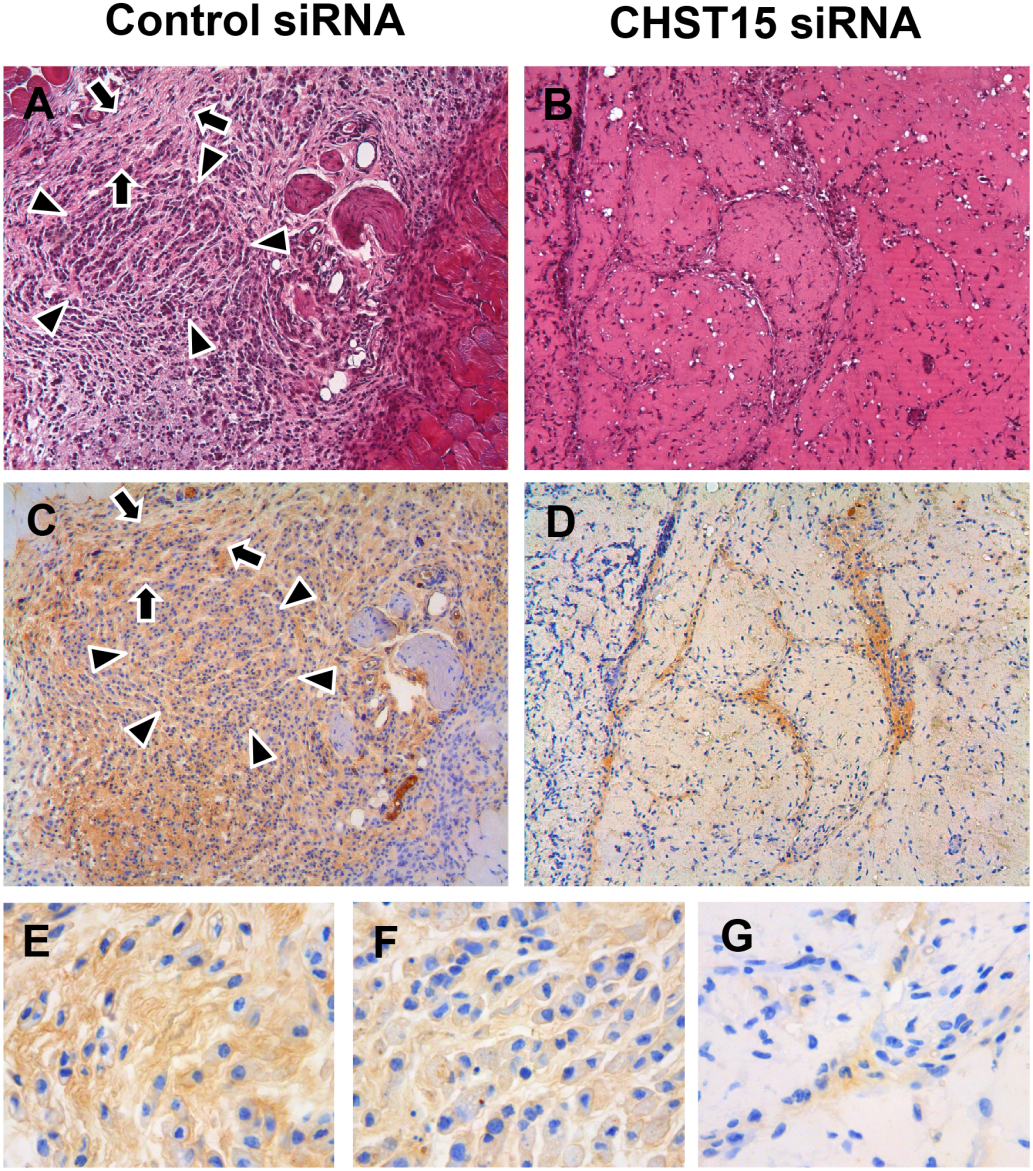
Effect of CHST15 siRNA on tumor histology in a xenograft model. (A, B) Tumors in nude mice at day 19 were stained with HE (original magnification ×100). Representative pictures were shown. Widespread necrosis lesions were prominent in the CHST15 siRNA treated mice (A) compared to the control siRNA treated mice (B). (C, D, E, F, G) Immunohistochemical staining of CHST15 (brown, original magnifications ×100 for C, D, x400 for E, F, G) in xenografts at day 19. Representative pictures were shown. Although almost all tumor cells in xenograft were positive for CHST15 in control siRNA treated mice (D), strong positive signal was observed in the invasive front rather than the center of tumor. CHST15-highly positive tumor cells exhibited mesenchymal-like morphology (E) while CHST15-low positive cells exhibited cord-like morphology (F), In contrast, small number of remnant tumor cells was weakly positive for CHST15 in CHST15 siRNA treated mice (C).

In order to demonstrate the efficacy of CHST15 siRNA, the pancreatic tumors in the nude mice were analyzed by HE staining and CHST15 immunohistochemical staining (Fig 3). In HE staining, widespread necrosis was observed in the CHST15 siRNA group (Fig 3A) compared to the control siRNA group (Fig 3B). Localization of CHST15 in the xenograft was evaluated by immunostaining against human CHST15 protein. CHST15 was highly expressed by tumor cells located in the invasive front (Fig 3A and 3C). Strong CHST15-staining was detected in the cytoplasm of cells with mesenchymal-like morphology (Fig 3E). In the center of tumor, the expression of CHST15 was relatively lower compared to the invasive front (Fig 3A and 3C). Weak localization of CHST15 was detectable in the cells of cord-like structure (Fig 3F). In contrast, a clear loss of CHST15 staining in the tumor regions of the CHST15 siRNA group (Fig 3D and 3G) was revealed.

### CS-E promoted the proliferation of PANC-1 cells in the presence of HGF

**Fig 4:**
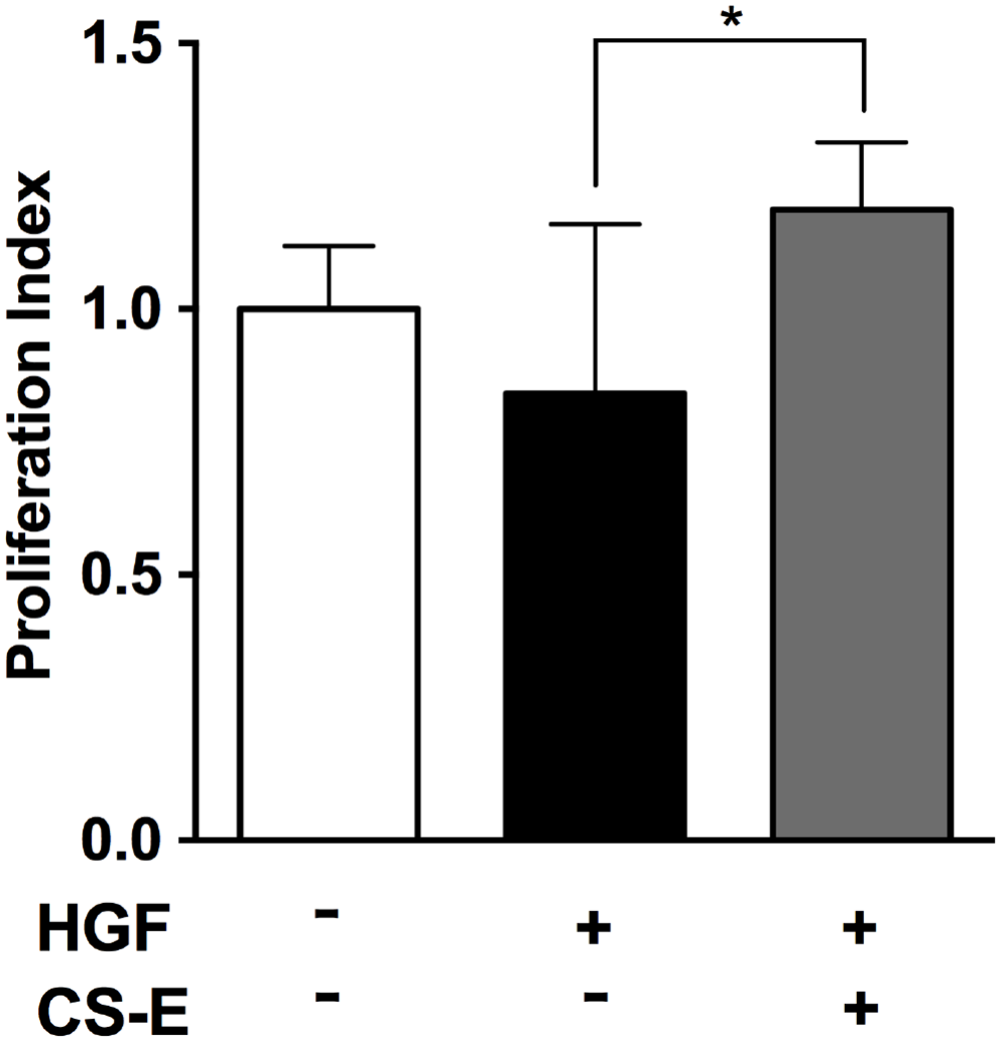
Effect of CS-E on PANC-1 tumor cell proliferation. A WST-1 assay was performed using PANC-1 cell. The mean level of proliferation in PANC-1 cells without adding CS-E and HGF (white column) was expressed as a standard (1.0) and the data were shown relative to the standard as a proliferation index. The proliferation index in PANC-1 cells treated with low dose HGF (black column) did not show statistical difference in the culture system. In contrast, the proliferation index in HGF-treated PANC-1 cells significantly increased when adding CS-E (gray column). Data were expressed as mean±SD (n = 6 in each).

The effect of CS-E on the proliferation of PANC-1 cells was assessed *in vitro* (Fig 4). Although HGF is known to promote proliferation of tumor cells, the lower dose of HGF used in this study did not induce significant tumor cell proliferation. However, the proliferation index of PANC-1 was significantly increased with the same dose of HGF in the presence of CS-E (Fig 4).

## Discussion

The prognosis for PDAC patients is still dismal, and revolutionary therapeutic options are strongly required. It is known that the high mortality associated with PDAC is mainly attributed to its peerless aggressiveness due to its early invasion and distant metastases. These malignant characteristics are observed in other advanced tumors like ovarian cancer. In ovarian cancer, CS-E is detected in both tumor and stromal cells and is involved in the peerless aggressiveness of the cancer and correlates with poor prognosis [21]. Based on their similar distribution patterns, it is thought that both tumor cell-derived as well as stromal cell-derived CS-E play vital roles in the various steps of tumor progression in advanced tumors.

To investigate the role of CS-E in PDAC, we used CHST15 siRNA, which selectively inhibits the expression of the CHST15 gene and the synthesis of CS-E. We chose this method because the selective inhibition of CS-E using a sufficient amount of chemical inhibitors or antibodies is still a challenge *in vivo*. We focused on the effect of CHST15 directly on tumor, but not stromal, cell proliferation, a primary step of tumor progression, in the human pancreatic cancer cell line PANC-1. In tumor proliferation, GAGs have been reported to act as co-receptors for soluble tumor growth factors. GAGs facilitate the formation of ligand-receptor complexes and activation of receptor signaling.

Because HGF is one of the key soluble factors produced by tumor cells as well as stromal cells in PDAC, we tested whether the activity of HGF could be increased in the presence of CS-E. Even when using a lower concentration of HGF, which failed to induce tumor cell proliferation, a significant increase in proliferation was confirmed in the presence of CS-E (Fig 4). CS-E alone did not induce tumor cell proliferation at the tested concentration, indicating that CS-E augments receptor signaling for HGF.

Unexpectedly, CHST15 siRNA alone directly inhibited the proliferation of PANC-1 cells *in vitro*. A distinct mechanism from co-receptors was also suggested. Although we did not examine the detailed kinetics during culture, levels of the cell cycle inhibitor-related gene p21^CIP1/WAF1^ increased in PANC-1 cells [22] that displayed a significant reduction in CHST15 mRNA levels at 48 h, suggesting that CHST15 gene silencing modified upstream pathways of p21. In this respect, up-regulation of p21 has been reported in some conditions in pancreatic cancer cells. For example, blocking Hedgehog signaling induced up-regulation of p21 [23, 24]. Because the CS-GAGs-Hedgehog interaction has been reported to enhance Hedgehog signaling [25], one possibility is that blocking CS-E synthesis could contribute to inhibiting Hedgehog signaling, which would lead to an up-regulation of p21. Further investigation is needed to understand the CHST15-mediated mechanisms regulating cancer cell proliferation related to the p21 pathway.

We also tested the effect of CHST15 siRNA on tumor growth by intratumoral injection in a xenograft model. After establishing the tumor, a single injection of CHST15 siRNA significantly suppressed further tumor growth (Fig 2A). We did not observe significant reduction of human CHST15 mRNA levels one week after CHST15 siRNA injection, at the time of sacrifice, compared to negative control siRNA. In contrast, histological examination of the xenograft clearly showed the reduction of human CHST15 protein-positive tumor cells by CHST15 siRNA one week after the single injection, indicating that successful inhibition of CHST15 was obtained. One possible explanation for the mRNA-protein imbalance is that the level of tumor-derived human CHST15 mRNA reached a peak at earlier time points and decreased thereafter to day 9, after which the efficacy on mRNA could not be detected sufficiently. Another possibility is that the critical level of CHST15 mRNA is not sufficiently detected only by PCR method. Because CHST15-highly expressed tumor cells can only be detected in the invasive front, but not in the center of xenograft, it is considered that *in vivo* CHST15 siRNA mainly acts on these tumor cells located in the invasive front. The number of these cells is limited among whole tumor cells and the mRNA signals might not be sufficiently detected by PCR that used whole xenograft sample. Once the initiation of tumor growth was suppressed by the CHST15 siRNA, and, subsequently, the production of CHST15 protein was inhibited, further growth signals provided by CHST15-mediated growth factors might have been effectively suppressed. Further studies to assess the time course and/or repeated siRNA injections could clarify the *in vivo* mechanism underlying the mRNA-protein relationship.

The present study showed, for the first time, that CHST15 siRNA could suppress tumor growth, which is considered as a primary stage of tumor progression at the primary site. However, there were several limitations in the present study. First, we used only PANC-1 cells in vivo although, we used three different cell lines in vitro and observed similar effects of CHST15 siRNA in tumor cell proliferation. Second, we used a skin xenograft model to simply examine the effect of CHST15 siRNA on the proliferation and growth of tumor cells. Orthotropic xenograft models will provide further information on the role of CHST15 siRNA in tumor cell proliferation in a tumor microenvironment. It will be worth attempting to clarify the whole picture of the role of CHST15 and CS-E in PDAC progression by analyzing cell-specific and stage-specific characteristics.

The route of siRNA delivery was limited to an intratumoral injection in the present study. However, we consider that the local injection has some advantages when dealing with hypovascular tumors as in PDAC and for siRNA whose systemic delivery is still a challenge. Actually, the intratumoral injection of siRNAs is currently available in clinical settings. An endoscopic ultrasound (EUS) is not only a diagnostic tool but also an interventional and therapeutic procedure. The evidence that interventional EUS is useful for PDAC treatment via injection therapy including siRNAs has been steadily accumulating [26–30]. Interventional EUS will be an essential part of the multidisciplinary approach to cancer treatment in the near future. This technique provides the ability to treat PDAC in a direct and relatively minimally invasive manner, with a very low incidence of procedural-related complications. We consider that the clinical application of CHST15 siRNA using EUS-fine needle injection for PDAC patients will be feasible in an actual clinic.

In summary, our study demonstrated that RNAi-mediated down-regulation of CHST15 effectively inhibits the proliferation and growth of pancreatic tumor cells. The CHST15 signaling pathway plays an important role in PDAC proliferation and growth and might serve as a potential therapeutic target for PDAC. Further studies are needed to clarify the effect and risk of CHST15 gene silencing by CHST15 siRNA in PDAC development and to enable its use in any clinical applications.

## Supporting Information

S1 Database Gene-suppressive effect of CHST15 siRNA on day-9 tumor in a xenograft model. Relative quantities of CHST15 mRNA in control siRNA treated and CHST15 siRNA treated xenografts at day 9. Data were expressed as mean±SD (Control siRNA: n = 7, CHST15 siRNA; n = 5).
(DOCX)
