# Supplementary material for: Inhibition of Cell Proliferation and Growth of Pancreatic Cancer by Silencing of Carbohydrate Sulfotransferase 15 In Vitro and in a Xenograft Model
Source: PLoS One. 2015 Dec 7;10(12):e0142981. doi: 10.1371/journal.pone.0142981 (PMC4671730; doi:10.1371/journal.pone.0142981)
Supplement: S1 Database — Relative quantities of CHST15 mRNA in control siRNA treated and CHST15 siRNA treated xenografts at day 9. Data were expressed as mean±SD (Control siRNA: n = 7, CHST15 siRNA; n = 5). (DOCX) [file pone.0142981.s001.docx]

CHST15 mRNA

　　　　　　　　
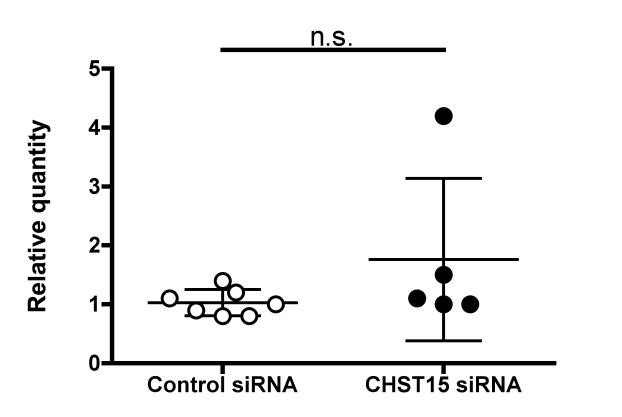


**Supporting Information 1.**

Gene-suppressive effect of CHST15 siRNA on day-9 tumor in a xenograft model. Relative quantities of CHST15 mRNA in control siRNA treated and CHST15 siRNA treated xenografts at day 9. Data were expressed as mean±SD (Control siRNA: n=7, CHST15 siRNA; n=5).
